# Supplementary material for: High-Dose Fenofibrate Stimulates Multiple Cellular Stress Pathways in the Kidney of Old Rats
Source: Int J Mol Sci. 2024 Mar 6;25(5):3038. doi: 10.3390/ijms25053038 (PMC10932055; doi:10.3390/ijms25053038)
Supplement: Supplementary file 1 [file ijms-25-03038-s001.zip › Supplementary Figure S1.pdf]

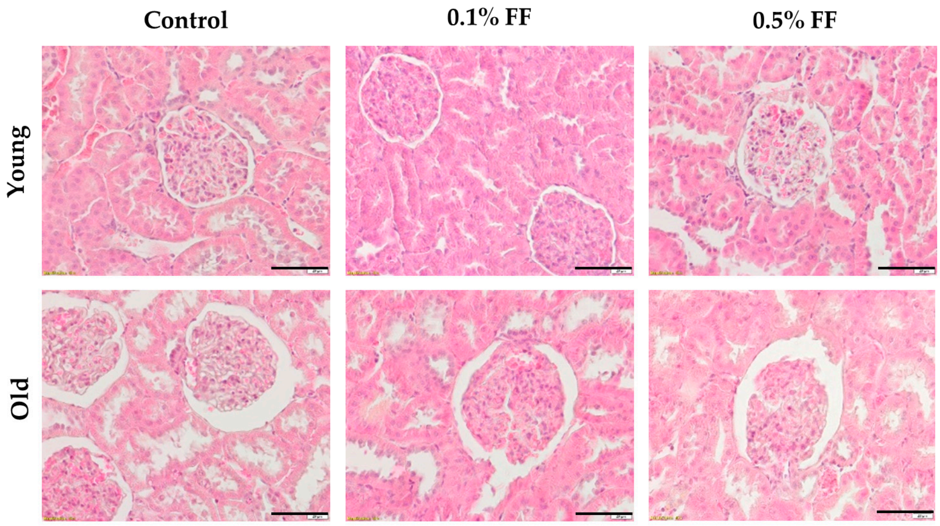

Figure S1: Effects of fenofibrate on the kidney morphology in young and old rats - hematoxylin and eosin staining (scale bar 50 μm)
