# Supplementary material for: High-Dose Fenofibrate Stimulates Multiple Cellular Stress Pathways in the Kidney of Old Rats
Source: Int J Mol Sci. 2024 Mar 6;25(5):3038. doi: 10.3390/ijms25053038 (PMC10932055; doi:10.3390/ijms25053038)
Supplement: Supplementary file 1 [file ijms-25-03038-s001.zip › Supplementary Table S1 PCR array data.pdf]

Supplementary Table S1: PCR array for stress and toxicity-associated genes (PARN-003ZA, Qiagen). Differentially expressed genes downregulated at least two-fold are printed in red, while the genes upregulated by at least two-fold are printed in green. Young (y) and old (o) rats treated with 0.1% (y.1, o.1) or 0.5% FF (y.5, o.5) were tested against their age controls. Measurements were performed on cDNA spools of 5 animals *per* group.

Symbol	Description	Molecular pathway	y.1FF /yC	y.5FF /yC	o.1FF /oC	o.5FF /oC
**Fth1**	Ferritin, heavy polypeptide 1	Oxidative Stress	1,12	-1,53	1,01	-1,63
**Gclc**	Glutamate-cysteine ligase, catalytic subunit	Oxidative Stress	-2,18	-2,90	-1,46	-3,67
**Gclm**	Glutamate cysteine ligase, modifier subunit	Oxidative Stress	-1,65	-1,49	-1,68	-2,80
**Gsr**	Glutathione reductase	Oxidative Stress	-1,59	-1,82	-1,27	-1,54
**Gstp1**	Glutathione S-transferase pi 1	Oxidative Stress	-2,31	-1,75	-1,35	-1,38
**Hmox1**	Heme oxygenase (decycling) 1	Oxidative Stress	-1,21	-1,82	1,28	3,23
**Hspa1b**	Heat shock 70kD protein 1B (mapped)	Oxidative Stress	-1,31	9,30	-6,14	6,76
**Nqo1**	NAD(P)H dehydrogenase, quinone 1	Oxidative Stress	-1,68	-1,28	-1,42	1,31
**Prdx1**	Peroxiredoxin 1	Oxidative Stress	-1,41	-1,61	-1,23	-1,20
**Sqstm1**	Sequestosome 1	Oxidative Stress	-1,51	-1,74	-1,39	-1,31
**Txn1**	Thioredoxin 1	Oxidative Stress	-1,40	-1,44	-1,17	-1,26
**Txnrd1**	Thioredoxin reductase 1	Oxidative Stress	-1,96	-2,06	-1,74	-1,12
**Adm**	Adrenomedullin	Hypoxia Signalling	-1,16	1,16	-1,31	2,10
**Arnt**	Aryl hydrocarbon receptor nuclear translocator	Hypoxia Signalling	-1,92	1,27	-2,20	1,90
**Bnip3l**	BCL2/adenovirus E1B interacting protein 3-like	Hypoxia Signalling	-1,50	-2,04	-1,32	-1,48
**Car9**	Carbonic anhydrase 9	Hypoxia Signalling	-1,87	5,92	-1,11	10,15
**Epo**	Erythropoietin	Hypoxia Signalling	-2,28	2,85	-2,19	8,95
**Ldha**	Lactate dehydrogenase A	Hypoxia Signalling	-1,08	-1,12	1,05	1,17
**Mmp9**	Matrix metallopeptidase 9	Hypoxia Signalling	-1,99	1,28	-1,17	5,00
**Serpine1 (Pai-1)**	Serpin peptidase inhibitor, clade E (nexin, plasminogen activator inhibitor type 1), memb. 1	Hypoxia Signalling	-1,91	1,66	-2,15	3,37

**Slc2a1**	Solute carrier family 2 (facilitated glucose transporter), member 1	Hypoxia Signalling	-2,33	-1,92	-1,39	-1,43
**Vegfa**	Vascular endothelial growth factor A	Hypoxia Signalling	-2,49	-1,13	-2,50	1,03
**Aqp1**	Aquaporin 1	Osmotic Stress	-2,10	-1,78	-1,76	-1,61
**Aqp2**	Aquaporin 2 (collecting duct)	Osmotic Stress	-1,40	1,14	-1,55	-1,45
**Aqp4**	Aquaporin 4	Osmotic Stress	-1,77	2,14	-1,96	2,29
**Cftr**	Cystic fibrosis transmembrane conductance regulator homolog (human)	Osmotic Stress	1,92	30,15	-4,54	6,55
**Edn1**	Endothelin 1	Osmotic Stress	-2,20	1,43	-1,68	2,70
**Hspa4l**	Heat shock protein 4	Osmotic Stress	-1,41	1,02	-1,33	2,00
**Nfat5**	Nuclear factor of activated T-cells 5	Osmotic Stress	-1,71	1,86	-1,72	2,88
**Slc5a3**	Solute carrier family 5 (sodium/myo-inositol cotransporter), member 3	Osmotic Stress	-3,10	-2,25	1,32	-2,33
**Slc9a3**	Solute carrier family 9 (sodium/hydrogen exchanger), member 3	Osmotic Stress	-3,28	1,58	-2,08	3,23
**Ccl12**	Chemokine (C-C motif) ligand 12	Inflammatory Response	-1,02	12,21	-3,96	8,52
**Cd40lg**	CD40 ligand	Inflammatory Response	1,08	8,19	-3,25	8,72
**Crp**	C-reactive protein, pentraxin-related	Inflammatory Response	-3,02	2,88	-4,62	7,86
**Ifng**	Interferon gamma	Inflammatory Response	-1,11	18,08	-8,22	5,81
**Il1a**	Interleukin 1 alpha	Inflammatory Response	-1,02	-1,23	-1,20	-1,24
**Il1b**	Interleukin 1 beta	Inflammatory Response	1,59	2,24	-2,48	4,32
**Il6**	Interleukin 6	Inflammatory Response	3,68	9,81	-4,04	6,21
**Tlr4**	Toll-like receptor 4	Inflammatory Response	1,18	3,49	-1,87	5,14
**Tnf**	Tumor necrosis factor (TNF superfamily, member 2)	Inflammatory Response	-2,99	1,85	-2,09	6,18
**Cdkn1a (Cip1, Waf1)**	Cyclin-dependent kinase inhibitor 1A	Cell Cycle Arrest & Checkpoints	-1,06	-2,05	-1,05	-1,14
**Chek1**	CHK1 checkpoint homolog (S. pombe)	Cell Cycle Arrest & Checkpoints	-1,67	6,34	-2,32	5,69
**Chek2**	CHK2 checkpoint homolog (S. pombe)	Cell Cycle Arrest & Checkpoints	-1,57	3,17	-2,15	4,59

**Ddit3 (Gadd153, Chop)**	DNA-damage inducible transcript 3	Cell Cycle Arrest & Checkpoints	-2,45	-1,92	-1,44	1,47
**Hus1**	HUS1 checkpoint homolog (S. pombe)	Cell Cycle Arrest & Checkpoints	-1,49	1,13	-1,45	2,11
**Mre11a**	meiotic recombination 11 homolog A (S. cerevisiae)	Cell Cycle Arrest & Checkpoints	-1,63	2,08	-2,60	3,39
**Nbn (Nbs1)**	Nibrin	Cell Cycle Arrest & Checkpoints	-1,08	1,23	1,32	1,34
**Rad17**	RAD17 homolog (S. pombe)	Cell Cycle Arrest & Checkpoints	-1,45	-1,17	-1,38	1,37
**Rad9**	RAD9 homolog (S. pombe)	Cell Cycle Arrest & Checkpoints	-1,87	1,15	-1,76	1,52
**Atm**	Ataxia telangiectasia mutated homolog (human)	Other DNA Damage Responses	-1,45	2,57	-2,21	3,18
**Ddb2**	Damage specific DNA binding protein 2	Other DNA Damage Responses	-1,60	-1,07	-1,99	1,48
**Gadd45a**	Growth arrest and DNA-damage-inducible, alpha	Other DNA Damage Responses	-1,24	-1,19	-1,31	-1,26
**Gadd45g**	Growth arrest and DNA-damage-inducible, gamma	Other DNA Damage Responses	-1,52	2,01	-3,54	-1,08
**Rad51**	RAD51 homolog (RecA homolog, E. coli) (S. cerevisiae)	Other DNA Damage Responses	-1,32	5,68	-2,63	5,66
**Tp53 (p53)**	Tumor protein p53	Other DNA Damage Responses	-1,40	-1,15	-1,36	1,08
**Xpc**	Xeroderma pigmentosum, complementation group C	Other DNA Damage Responses	-1,64	-1,12	-1,85	1,01
**Casp1 (Ice)**	Caspase 1	Apoptosis	1,11	2,25	-1,60	2,55
**Casp7**	Caspase 7	Apoptosis	-1,78	-2,00	-1,46	1,25
**Fas**	Fas (TNF receptor superfamily, member 6)	Apoptosis	1,04	1,25	-1,26	1,08
**Mcl1**	Myeloid cell leukemia sequence 1	Apoptosis	-1,29	-2,50	-1,04	-1,36
**Tnfrsf10b**	G03 Rn.105558 NM_001108873 Tumor necrosis factor receptor superfamily, memb. 10b	Apoptosis	-1,44	-3,18	-1,44	-1,32
**Tnfrsf1a (Tnfr1)**	Tumor necrosis factor receptor superfamily, member 1a	Apoptosis	-1,45	-1,29	-2,14	1,10
**Grb2**	Growth factor receptor bound protein 2	Necrosis	-1,54	-1,82	-1,39	-1,02
**Parp1 (Adprt1)**	Poly (ADP-ribose) polymerase 1	Necrosis	-1,29	-1,23	-1,10	1,27
**Parp2**	Poly (ADP-ribose) polymerase 2	Necrosis	-1,18	1,16	-1,78	1,51
**Pvr**	Poliovirus receptor	Necrosis	-3,11	-1,74	-2,13	2,62

**Ripk1**	Receptor (TNFRSF)-interacting serine-threonine kinase 1	Necrosis	-2,00	-1,15	-1,55	1,30
**Ripk3**	Receptor-interacting serine-threonine kinase 3	Necrosis	-1,52	1,62	-1,85	2,58
**Txnl4b**	Thioredoxin-like 4B	Necrosis	-1,67	1,18	-1,78	2,04
**Atg12**	ATG12 autophagy related 12 homolog (S. cerevisiae)	Autophagy	-1,12	-1,26	-1,29	1,26
**Atg7**	ATG7 autophagy related 7 homolog (S. cerevisiae)	Autophagy	-1,46	1,02	-1,31	1,73
**Becn1**	Beclin 1, autophagy related	Autophagy	-1,34	-1,11	-1,75	-1,46
**Ulk1**	Unc-51 like kinase 1 (C. elegans)	Autophagy	-1,94	1,13	-1,98	1,63
**Atf4**	Activating transcription factor 4 (tax-responsive enhancer element B67)	Unfolded Protein Response	-2,19	-1,46	-1,53	1,08
**Atf6**	Activating transcription factor 6	Unfolded Protein Response	-1,69	1,89	-2,83	1,79
**Bbc3**	Bcl-2 binding component 3	Unfolded Protein Response	-1,71	-1,24	-2,19	1,04
**Bid**	BH3 interacting domain death agonist	Unfolded Protein Response	-1,71	-1,00	-1,63	2,57
**Calr**	Calreticulin	Unfolded Protein Response	-1,91	-1,47	-1,49	-1,47
**Dnajc3**	DnaJ (Hsp40) homolog, subfamily C, member 3	Unfolded Protein Response	-1,31	-1,06	-1,31	-1,04
**Hsp90aa1**	Heat shock protein 90, alpha (cytosolic), class A member 1	Unfolded Protein Response	-1,55	-1,75	1,00	1,50
**Hsp90b1**	Heat shock protein 90, beta, member 1	Unfolded Protein Response	-1,87	-2,00	-1,29	-1,52
**Hspa4 (Hsp70)**	Heat shock protein 4-like	Unfolded Protein Response	-1,23	-1,51	-1,38	-1,12
**Hspa5 (Grp78)**	Heat shock protein 5	Unfolded Protein Response	-2,28	-2,76	-1,01	-1,55
**Xbp1**	X-box binding protein 1	Unfolded Protein Response	-2,07	-2,37	1,01	-1,57
**Hprt1**	Hypoxanthine phosphoribosyltransferase 1	*Housekeeping gene*	-1,03	-1,11	-1,03	-1,04
**Rplp1**	Ribosomal protein, large, P1	*Housekeeping gene*	1,03	1,11	1,03	1,04
