# Supplementary material for: High-Dose Fenofibrate Stimulates Multiple Cellular Stress Pathways in the Kidney of Old Rats
Source: Int J Mol Sci. 2024 Mar 6;25(5):3038. doi: 10.3390/ijms25053038 (PMC10932055; doi:10.3390/ijms25053038)
Supplement: Supplementary file 1 [file ijms-25-03038-s001.zip › Supplementary Table S2 List of primers.pdf]

Supplementary Table S2: List of primers.

Gene symbol	Gene name	Forward and reverse primer sequences
**Acox1**	Peroxisomal acyl-coenzyme A oxidase 1	GTCTCTTGTATTCTCTCTATGG<br>GTAAGATTCATGGACCTCTG
**Casp1**	Caspase 1	GCATTAAGAAGGCCCATATAGAGA<br>TGAAGAGAGATCCCTGGACAG
**Ccl12 (Mcp5)**	Chemokine (C-C motif) ligand 12	CCAGATTCAGTGTTCACCCCA<br>GGCTGCTTGTGATTTTCCTGT
**Cftr**	Cystic fibrosis transmembrane conductance regulator	ACCTCCCAACAGCTCAAACA<br>AAGGCTCGAAGTGTCCACAG
Cpt1	Carnitine palmitoyltransferase 1	ATGTTTGACCCAAAGCAGTACCCC<br>TCGCCTGCGATCATGTAGGAAAC
**Crp**	C-reactive protein	AATGTTTTGAACTGGCGGGC<br>TGCCTCAGGACTCACAACAG
**Edn1**	Endothelin 1	AGCGCGTCGTCCCGTATG<br>CTGTTCCCTTGGTCTGTGGT
**Hmgcs2**	3-hydroxy-3-methylglutaryl-CoA synthase 2	ACCACAAGGTGAACTTCTCTC<br>TTTGGGTAACGGCTCTGCTC
**Ho-1**	Heme oxygenase 1	TCAGAAGGGTCAGGTGTCCA<br>GGGTTCTGCTTGTTTCGCTC
**Hprt1**	Hypoxanthine phosphoribosyltransferase 1	TGCTGAAGATTTGGAAAAGGTGT<br>GGCCTCCCATCTCCTTCATG
**Il-1b**	Interleukin 1b	GCTACCTATGTCTTGCCCGT<br>CATCCCACGAGTCACAGAGG
**Il-6**	Interleukin 6	TGAGAAAAGAGTTGTGCAATGG<br>CCATCATTTCTTTGTATTTCTGGAAG
**Lcad (Acadl)**	Long-chain acyl-CoA dehydrogenase	TCGAGCAGTTTATCCCCCAG<br>TGAACACCTTGCTTCCATTGAG
**Mcad (Acadm)**	Medium chain acyl-CoA dehydrogenase	GCAGCTGATGATGTGTGCCTA<br>ATCTGGGTTAGATCGCGTCA
**Nlrp3**	NLR family pyrin domain containing 3	GGAGTGGATAGGTTTGCTGGG<br>GAACCTGCTTCTCACATGCC
**Nrf2 (Nfe2l2)**	Nuclear factor erythroid 2-related factor 2	CCCACCTTGAACACAGATTTCG<br>TAATGGCAGCAGAGGAAGGC
**Rplp1**	Ribosomal protein lateral stalk subunit P1	AAGCAGCTGGTGTCAATGTTG<br>CAGCCCCTACATTGCAGATGA
**Serpine1 (Pai-1)**	Serpin E1	CCGGCAGCAGATCCAAGAT<br>ATGACCCCATGAGCTCCTTG
**Sesn2**	Sestrin 2	CGTAGACACCTCCATGCTCC<br>CATTCTGCGGGTCGTCTTCT
**Sirt1**	Sirtuin 1	CAGAACCACCAAAGCGGAAAAA<br>GAAACCCCAGCTCCAGTCAGAA
**Slc9a3**	Solute carrier family 9 member A3	GGACATCTCAGGACAAATTGGAC<br>GAGCAGATCTTCTCATGAGGACT
**Srxn1**	Sulfiredoxin 1	CCCAAGGCGGTGACTACTAC<br>GACCTCACGAGCTTGGCAG
**Vegfa**	Vascular endothelial growth factor A	CATGCGGATCAAACCTCACC<br>TGTTCTATCTTTCTTTGGTCTGCA
