# Supplementary figures and images for: High-Dose Fenofibrate Stimulates Multiple Cellular Stress Pathways in the Kidney of Old Rats
Source: Int J Mol Sci. 2024 Mar 6;25(5):3038. doi: 10.3390/ijms25053038 (PMC10932055; doi:10.3390/ijms25053038)

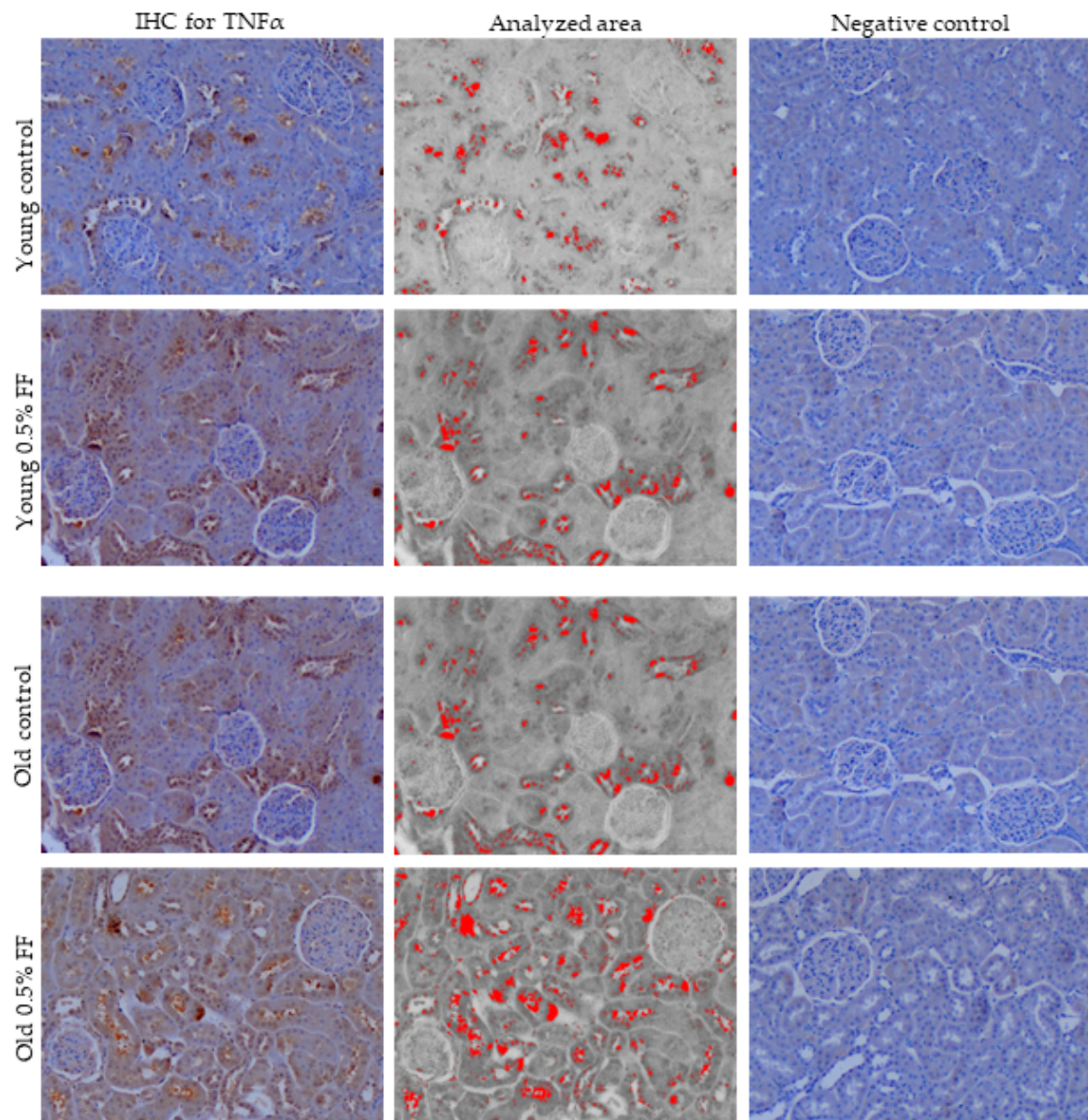

**Figure S2:** Immunohisto- chemical staining for TNF $\alpha$  – examples of image analyses.

Supplement: Supplementary file 1 [file ijms-25-03038-s001.zip › Supplementary Figure S2.pdf]
